# Phylogeography and population structure of the grape powdery mildew fungus, *Erysiphe necator*, from diverse *Vitis *species

**DOI:** 10.1186/1471-2148-10-268

**Published:** 2010-09-01

**Authors:** Marin Talbot Brewer, Michael G Milgroom

**Affiliations:** 1 Department of Plant Pathology and Plant-Microbe Biology, Cornell University, Ithaca, NY 14853, USA

## Abstract

**Background:**

The grape powdery mildew fungus, *Erysiphe necator*, was introduced into Europe more than 160 years ago and is now distributed everywhere that grapes are grown. To understand the invasion history of this pathogen we investigated the evolutionary relationships between introduced populations of Europe, Australia and the western United States (US) and populations in the eastern US, where *E. necator *is thought to be native. Additionally, we tested the hypothesis that populations of *E. necator *in the eastern US are structured based on geography and *Vitis *host species.

**Results:**

We sequenced three nuclear gene regions covering 1803 nucleotides from 146 isolates of *E. necator *collected from the eastern US, Europe, Australia, and the western US. Phylogeographic analyses show that the two genetic groups in Europe represent two separate introductions and that the genetic groups may be derived from eastern US ancestors. Populations from the western US and Europe share haplotypes, suggesting that the western US population was introduced from Europe. Populations in Australia are derived from European populations. Haplotype richness and nucleotide diversity were significantly greater in the eastern US populations than in the introduced populations. Populations within the eastern US are geographically differentiated; however, no structure was detected with respect to host habitat (i.e., wild or cultivated). Populations from muscadine grapes, *V. rotundifolia*, are genetically distinct from populations from other *Vitis *host species, yet no differentiation was detected among populations from other *Vitis *species.

**Conclusions:**

Multilocus sequencing analysis of the grape powdery mildew fungus is consistent with the hypothesis that populations in Europe, Australia and the western US are derived from two separate introductions and their ancestors were likely from native populations in the eastern US. The invasion history of *E. necator *follows a pattern consistent with plant-mediated dispersal, however, more exhaustive sampling is required to make more precise conclusions as to origin. *E. necator *shows no genetic structure across *Vitis *host species, except with respect to *V. rotundifolia*.

## Background

Introduced pathogens have led to devastating epidemics in naïve host populations that lack evolved defences, as demonstrated by the plant pathogen *Cryphonectria parasitica*, the fungus that causes chestnut blight. Its introduction from Asia [1] practically eliminated the American chestnut (*Castanea dentata*) and markedly altered the species composition of forests throughout eastern North America. Source pathogen populations are expected to be more diverse than introduced populations because introduced populations have smaller effective population sizes due to losses in genetic diversity from population bottlenecks and genetic drift associated with small founder population sizes [2,3]. However, this pattern could be reversed if multiple divergent lineages from separate sources colonize an area [4,5]. Where introductions are few, haplotypes in introduced populations should be a subset of those in the source population [6,7]. Additionally, for sexually reproducing organisms, recombination from sexual reproduction may be more prevalent in source or native populations, whereas clonal reproduction may dominate in introduced or marginal populations since multiple mating types necessary for sexual reproduction may not be present [8-10]. However, lack of variation in introduced populations can make it difficult to detect recombination.

The focus of this research is the invasion history and population structure of the grape powdery mildew fungus, *Erysiphe necator *(formerly *Uncinula necator*), an obligate parasite of *Vitis *species that was introduced into Europe and, eventually, all other wine-producing regions of the world. Historical records support the hypothesis that the source of the introduction is eastern North America [11]. Powdery mildew was described on grapes in North America in 1834, prior to its discovery in Europe in 1845 [12]. Eastern North America is the centre of origin for many wild species of *Vitis *that have relatively high levels of resistance to many diseases and pests of grapevines, including powdery mildew [13,14]. After its introduction to Europe, grape powdery mildew was observed throughout all wine-producing regions of the world, including California in 1859 [15] and Australia in 1866 [16]. *E. necator *most likely dispersed long distances by the movement of grapevines, which were frequently traded between continents in the mid-1800's and later. *E. necator *remains dormant as mycelium in dormant buds, or as sexual spores in cleistothecia in the bark of vines [17,18].

Population genetic studies on *E. necator *to date have been limited to introduced populations in Europe and Australia where two distinct, yet sympatric, genetic groups have been consistently found [19-25]. The groups, designated as A and B (or groups I and III in earlier studies), were originally identified using anonymous markers assayed by RAPDs, ISSRs and AFLPs. Subsequent gene sequence analysis detected fixed nucleotide differences between groups at several nuclear loci, including 14 α-demethylase (*CYP51*), the internal transcribed spacer (*ITS*) regions of ribosomal DNA (rDNA) [26], and beta-tubulin (*TUB2*) [27]. In India, a third genetic group was found, defined by RAPDs and a unique *ITS *sequence [19,26]. Small differences in reproductive fitness [25] and temporal variation have been found between groups A and B [22,23,25,26] leading to the hypothesis that temporal variation between the groups may be maintaining the differentiation by preventing interbreeding [28]. Group A is genetically less diverse than group B, thus it has been suggested that it is clonal, whereas group B is sexually reproducing [19,23]. Groups A and B produce viable sexual progeny (ascospores) in laboratory crosses [21,22,29], but recombinants have not been found in nature.

We had two major objectives for this study. First, to understand the evolutionary processes that led to the existence of groups A and B of *E. necator *in introduced populations, we tested the hypothesis that A and B were derived from separate introductions, as opposed to diverging after their introduction. To address this question, it was essential to study the population structure in eastern North America, the putative source population. Because no information was available on the population genetics of *E. necator *in North America, our major second objective was to describe the diversity and population structure in the eastern Unites States (US). We tested the hypothesis that if the eastern US population was a potential source of introductions, haplotypes found in introduced populations of Europe, Australia, and the western US would also be found in the eastern US. Moreover, we predicted that populations in the eastern US would have greater haplotype and nucleotide diversity than introduced populations. Finally, we tested the hypotheses that the population in the eastern US is structured by geography, *Vitis *host species, or host habitat (wild or cultivated *Vitis*).

## Results

### Genetic diversity in eastern US and introduced populations

We obtained 146 isolates of *E. necator *from diverse wild and cultivated *Vitis *species collected from the eastern US (northeast, southeast and central) and from cultivated *V. vinifera *from the western US, Europe, and Australia (Table 1; Additional file 1: Table S1). We also collected isolates of powdery mildew (*E. necator *var. *ampelopsidis *[30]) from *Parthenocissus quinquefolia*. We sequenced a total of 1803 nucleotides from three nuclear gene regions: the internal transcribed spacer and the intergenic spacer regions of nuclear rDNA (*ITS/IGS*), beta-tubulin (*TUB2)*, and translation elongation factor 1-α (*EF1-α*). We were unable to amplify *IGS *from isolates sampled from *P. quinquefolia*. However, *E. necator *var. *ampelopsidis *from *P. quinquefolia *is markedly divergent with 94.9%, 93.0%, and 91.7% similarity to the consensus sequence of isolates from *Vitis *spp. for *ITS, TUB2*, and *EF1-α*, respectively. For comparison, the lowest sequence similarity within *E. necator *from *Vitis *spp. was 99.8%, 99.5%, and 99.4%. Among isolates of *E. necator *from *Vitis *spp. there were 37 segregating sites and 45 multilocus haplotypes (Table 2). *EF1-α *contained the most segregating sites, followed by *TUB2*, and *ITS/IGS*. All of the polymorphisms in *EF1-α *and *TUB2 *were found in introns or as synonymous substitutions in coding regions.

**Table 1:** *Vitis *host species, host habitats and geographic regions where *Erysiphe necator *was collected.

*Vitis *Host Species	Wild	Cultivated	Host Regions
*V. vinifera *(European wine grape)		71	southeast US, central US, northeast US, western US, Europe, Australia
Vinifera hybrids^1^		29	southeast US, central US, northeast US
*V. labrusca *and labrusca hybrids^2 ^(e.g., 'Concord')	7	9	southeast US, northeast US
*V. aestivalis*	13		southeast US, central US, northeast US
*V. riparia*	12		southeast US, central US, northeast US
*V. rotundifolia *(muscadine)^3^	2	3	southeast US

^1^vinifera hybrids refer to interspecific hybrids derived from crosses between the European wine grape, *V. vinifera*, and wild American *Vitis *species other than *V. labrusca*.

^2^labrusca hybrids (*i.e*. 'Concord' and 'Niagara'; sometimes referred to as *V. labruscana*) refer to *V. vinifera *x *V. labrusca *hybrids that are derived primarily from *V. labrusca *because of repeated backcrossing.

^3^Sometimes referred to as *Muscadinia rotundifolia*.

**Table 2:** Haplotypes and polymorphic sites among isolates of *Erysiphe necator *based on partial sequences of three gene regions.

Haplotype^1^	*N*	Polymorphic Sites^2^			Region (*N*)^3^	Host *Vitis *spp. (*N*)^4^
				
		*ITS/IGS*	*TUB2*	*EF1- α*		
*1 (aaa)*	16	TGGTGG/CGGTC	TGCCTTTATCCC	CTCGACCATTTCGC	SE (8), NE (8)	vin (8), hyb (3), aes (2), rip (3)
*2 (aab)*	4	....../.....	............	........C.....	NE (4)	vin (2), hyb (1), rip (1)
*3 (aac)*	1	....../.....	............	...........T..	NE (1)	hyb (1)
*4 (aad)*	1	....../.....	............	.........C....	SE (1)	aes (1)
*5 (aae)*	3	....../.....	............	.....A........	NE (3)	hyb (2), lab (1)
*6 (aaf)*	4	....../.....	............	.....A.G......	NE (4)	hyb (2), lab (1), rip (1)
*7 (aag)*	1	....../.....	............	TC............	NE (1)	rip (1)
*8 (aah)*	7	....../.....	............	TC...A......A.	SE (2), NE (5)	hyb (3), lab (1), aes (1), rip (2)
*9 (aai)*	6	....../.....	............	.............T	SE (1), NE (5)	vin (1), lab (1), aes (3), rip (1)
*10 (aaj)*	6	....../.....	............	......T......T	NE (6)	hyb (5), lab (1)
*11 (aak)*	5	....../.....	............	...T.........T	SE (3), C (1), NE (1)	vin (2), hyb (2), rip (1)
*12 (aal)*	2	....../.....	............	..........C..T	SE (1), NE (1)	vin (2)
*13 (aam)*	1	....../.....	............	....G.....C..T	NE (1)	vin (1)
*14 (aba)*	3	....../.....	..T.........	..............	SE (3)	vin (1), lab (1), aes (1)
*15 (abb)*	1	....../.....	..T.........	........C.....	SE (1)	lab (1)
*16 (abl)*	1	....../.....	..T.........	..........C..T	C (1)	hyb (1)
*17 (abn)*	1	....../.....	..T.........	............A.	C (1)	rip (1)
*18 (ach)*	1	....../.....	.C..........	TC...A......A.	C (1)	vin (1)
*19 (acl)*	2	....../.....	.C..........	..........C..T	C (2)	vin (1), hyb (1)
*20 (aco)*	1	....../.....	.C..........	..A.......C..T	C (1)	hyb (1)
*21 (adb)*	1	....../.....	C...........	........C.....	NE (1)	vin (1)
*22 (aeh)*	2	....../.....	.........T..	TC...A......A.	NE (2)	hyb (2)
*23 (afn)*	2	....../.....	.......T....	............A.	C (2)	vin (2)
*24 (agk)*	1	....../.....	..........T.	...T.........T	SE (1)	vin (1)
*25 (baa)*	3	....../....T	............	..............	SE (1), NE (2)	lab (3)
*26 (bab)*	2	....../....T	............	........C.....	NE (2)	vin (1), lab (1)
*27 (bai)*	1	....../....T	............	.............T	NE (1)	lab (1)
*28 (bal)*	1	....../....T	............	..........C..T	NE (1)	vin (1)
*29 (bba)*	1	....../....T	..T.........	..............	NE (1)	hyb (1)
*30 (bbl)*	1	....../....T	..T.........	..........C..T	NE (1)	hyb (1)
*31 (bha)*	2	....../....T	..T..A......	..............	NE (2)	lab (2)
*32 (bia)*	1	....../....T	......C.....	..............	NE (1)	lab (1)
*33 (cba)*	23	....../...CT	..T.........	..............	SE (8), EU (13), AU (2)	vin (18), hyb (1), aes (2), rip (2)
*34 (dba)*	1	....../..CCT	..T.........	..............	SE (1)	rot (1)
*35 (dja)*	4	....../..CCT	..TG........	..............	SE (4)	rot (4)
*36 (eba)*	1	....C./.....	..T.........	..............	NE (1)	vin (1)
*37 (fae)*	1	.T..../.....	............	.....A........	SE (1)	hyb (1)
*38 (gac)*	1	.....A/.....	............	...........T..	C (1)	hyb (1)
*39 (hac)*	1	..A.../.....	............	...........T..	SE (1)	aes (1)
*40 (ika)*	1	...A../....T	..T.C.......	..............	NE (1)	aes (1)
*41 (jai)*	18	C...../T....	............	.............T	W (12), EU (6)	vin (18)
*42 (jak)*	1	C...../T....	............	...T.........T	EU (1)	vin (1)
*43 (jli)*	3	C...../T....	...........T	.............T	W (1), EU (2)	vin (3)
*44 (jll)*	2	C...../T....	...........T	..........C..T	EU (2)	vin (2)
*45 (kmi)*	4	C...../TA...	........C...	.............T	AU (4)	vin (4)

^1^Numbers refer to the multilocus haplotypes; letters in parentheses refer to the haplotypes for *ITS/IGS *(the internal transcribed spacer of rDNA/the intergenic spacer of rDNA)*, TUB2 *(beta-tubulin), and *EF1-α *(translation elongation factor 1-α), respectively. Group A is represented by haplotype *33 *and group B is represented by haplotypes *41-45*.

^2^Only segregating sites are listed, which correspond to the following nucleotide positions in the referenced GenBank accessions: *ITS *(GQ255473; total length 591 nucleotides): 48, 84, 86, 170, 420, 462; *IGS *(GQ255476; 347 nt): 108, 206, 211, 216, 223; *TUB2 *(GQ255475; 442 nt): 24, 37, 79, 82, 128, 183, 207, 288, 316, 344, 356, 368; *EF1-α *(GQ255471; 423 nt): 2, 9, 25, 33, 102, 189, 210, 227, 228, 231, 336, 381, 384, 420.

^3^regions are: SE = southeast US, C = central US, NE = northeast US, W = western US, EU = Europe, AU = Australia. *N *= the number of isolates from each region with the designated haplotype.

^4^hosts are: vin = *V. vinifera*, hyb = vinifera hybrids, lab = *V. labrusca *and labrusca hybrids, aes = *V. aestivalis*, rip = *V. riparia*, rot = *V. rotundifolia. N *= the number of isolates from each host with the designated haplotype.

Based on measurements of Tajima's *D *[31], *ITS/IGS *and *EF1-α *do not deviate from neutral evolution (Table 3). However, *TUB2 *deviates significantly from neutrality in the eastern US population. Significant negative values for Tajima's *D *can result from population bottlenecks followed by rapid population expansion or from selective sweeps acting on or near the loci under investigation. Since this effect is not detected across the entire genome, as would be expected with demographic effects, this is suggestive that the deviation from neutrality in *TUB2 *is from selection.

**Table 3:** Haplotype richness, sequence diversity and neutrality estimates for populations of *Erysiphe necator*.

Locus	**Population**^**1**^	**Haplotype richness (*h***_**R**_)^**2**^	**Watterson's θ (θ**_**w**_)	π	Tajima's *D *(*P*-value)
*ITS/IGS*	Eastern US	**6 **(9)^3^	0.00123	0.00085	-1.167 (0.108)
	Introduced	**3**	0.00123	0.00217	1.901 (0.975)
		***P *= 0.013**	*P *= 0.485	*P *= 1.000	
					
*TUB2*	Eastern US	** 7 **(11)	**0.00314**	0.00159	**-1.598 (0.013)**
	Introduced	**4**	**0.00157**	0.00192	0.478 (0.727)
		***P *= 0.049**	***P *= 0.050**	*P *= 0.833	
					
*EF1-α*	Eastern US	**11 **(15)	**0.00601**	**0.00472**	-0.680 (0.270)
	Introduced	**4**	**0.00164**	**0.00142**	-0.281 (0.411)
		***P *< 0.001**	***P *= 0.001**	***P *= 0.001**	
					
Combined	Eastern US	** 21 **(40)	**0.00282**	0.00194	-
	Introduced	**6**	**0.00141**	0.00193	-
		***P *< 0.001**	***P *= 0.001**	*P *= 0.497	

^1^The introduced population comprises Europe, Australia and the western US; the eastern US comprises northeast, southeast and central US (Table 1).

^2^To account for sample size differences in eastern US (*N *= 103) and introduced populations (*N *= 43), we used rarefaction analysis [66] for haplotype richness, θ_w_, and π in the native population. Diversity estimates where the eastern US population is significantly more diverse than the introduced populations (*P *< 0.05) are in bold.

^3^Numbers in parentheses are the number of observed haplotypes among the 103 isolates from the eastern US population without correction by rarefaction analysis.

We estimated several population genetic parameters in the eastern US and introduced populations, including haplotype richness (*h*_R_), Watterson's theta (θ_w_), and pairwise nucleotide diversity (π). Haplotype richness is significantly greater in the eastern US than in the introduced populations for each locus and the multilocus haplotypes (Table 3), even when adjustments are made for differences in sample size (see Methods). Additionally, there is greater nucleotide polymorphism (θ_w_) in the eastern US population for *TUB2*, *EF1-α *and for all three loci combined. There is greater pairwise nucleotide diversity (π) for *EF1-α *in the eastern US population. However, π is greater in the introduced population for *ITS/IGS*.

### Phylogeography

**Figure 1:**
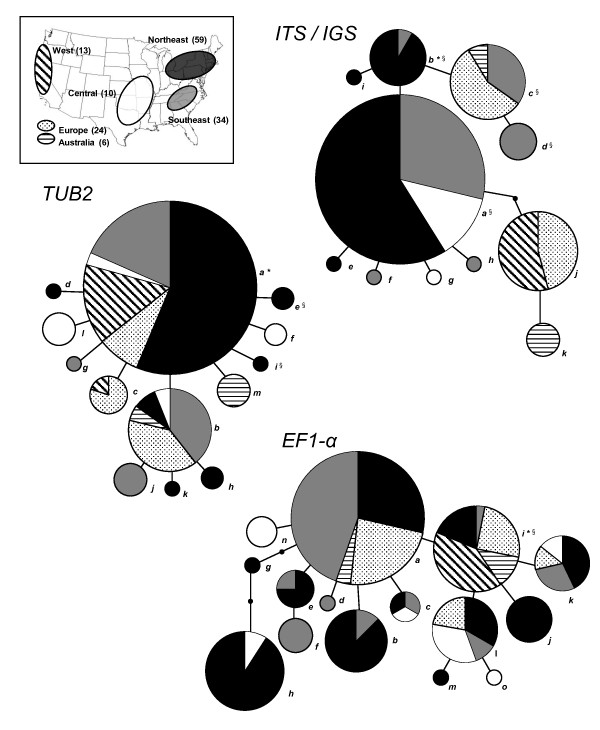
**Haplotype networks of *ITS/IGS, TUB2 *and *EF1-α ***. Networks constructed in TCS 1.21 [60,61]. Each haplotype is represented as a circle proportional in size to the number of isolates in each haplotype. Inferred intermediate haplotypes are represented by a small solid dot. Each line segment represents a single mutation. The letters defining haplotypes in Table 2 are shown to the right of each node. Geographic origins of isolates in each haplotype are proportionally represented in pie charts by different patterns shown in the key in the centre of the figure. The ancestral haplotypes determined by root probability [33] are indicated by asterisks (*), whereas those determined by maximum parsimony using ***E. necator ***var. ***ampelopsidis ***as the outgroup are indicated by §.

**Figure 2:**
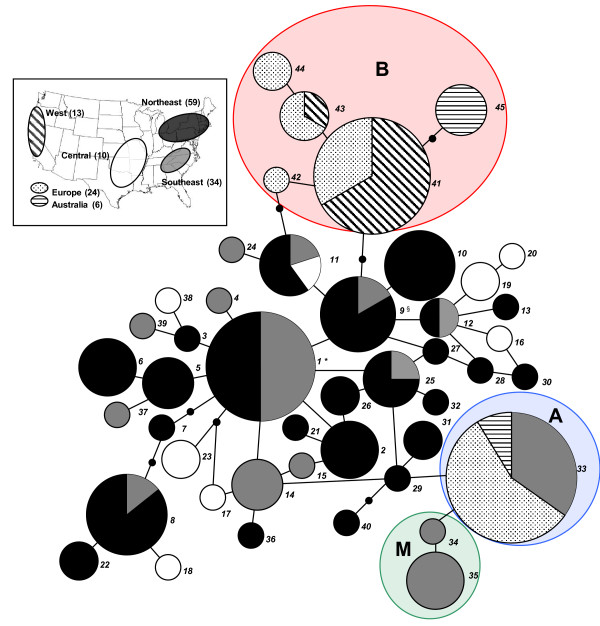
**Multilocus haplotype network for *Erysiphe necator***. Network constructed in TCS 1.21 [60,61]. Each haplotype is represented as a circle proportional in size to the number of isolates in each haplotype. Inferred intermediate haplotypes are represented by a small solid dot. Each line segment represents a single mutation. The numbers defining multilocus haplotypes in Table 2 are shown to the right of each node. Geographic origins of isolates in each haplotype are proportionally represented in pie charts by different patterns shown in the key in the upper left. The haplotypes that include group B isolates are enclosed in a magenta ellipse and marked with a 'B'; the haplotype that includes group A isolates is enclosed in a green ellipse and marked with an 'A'; and the haplotypes that include isolates from muscadine grapes (***V. rotundifolia***) are enclosed in a blue ellipse and are marked with an 'M'. The ancestral haplotype determined by root probability [33] is indicated by an asterisk (*), whereas that determined by maximum parsimony using ***E. necator ***var. ***ampelopsidis ***as an outgroup is indicated by §.

To determine evolutionary relationships among isolates, we constructed networks for the three gene regions and multilocus haplotypes (Figures 1 and 2). Ancestral haplotypes identified based on rooting probability [32,33] and maximum parsimony using *E. necator *var. *ampelopsidis *as an outgroup were from the eastern US in all cases. Based on maximum parsimony the outgroup haplotypes would be at least 23 mutational steps from the putative ancestors for *ITS/IGS*, 29 steps for *TUB2*, 35 steps for *EF1-α*, and at least 87 steps for the combined multilocus haplotype. Because of this degree of divergence TCS did not place the outgroup in the same network. The internal position of haplotypes from eastern North America is particularly noticeable for the multilocus network (Figure 2), whereas all haplotypes of isolates from introduced populations (represented by striped and stippled patterns) are at or near the tips of the network. Isolates from the western US have the same haplotypes (nos. *41 *and *43*) as isolates in group B from Europe (Table 2; Figures 1 and 2), which suggests that populations in the western US were introduced from Europe. In addition to the 13 isolates from the western US reported here, 17 isolates from California and one from Oregon had the same *IGS *sequence that is found only in group B (data not shown). We did not sequence additional loci for these isolates from the western US because all were like group B for *IGS*.

The majority of haplotypes for the individual loci, and especially for the multilocus network, are represented by individuals from the eastern US populations, demonstrating that populations in the eastern US are more diverse than in Europe, Australia and the western US. The sample size from the eastern US population is larger; however, this difference is accounted for in the comparisons of diversity estimates by rarefaction analyses. Although isolates from the central US are represented by diverse haplotypes, they are mostly derived haplotypes at or near the tips of the multilocus network, which suggests that this region is peripheral to the centre of diversity. All isolates obtained from *V. rotundifolia *belonged to two multilocus haplotypes (nos. *34 *and *35*; labelled 'M' in Figure 2) at the tips, derived from group A (haplotype no. *33*) and not shared by isolates from any other host species.

The haplotype networks show that genetic groups A and B from introduced populations are distinct from each other at all loci and are derived from North American ancestors (Figures 1 and 2). Because of these differences, and the internal position of North American haplotypes, groups A and B almost certainly represent two separate introductions instead of diverging after introduction. We found no genetic variation among group A isolates, and this same multilocus haplotype (no. *33*) was common in the southeastern US in isolates from diverse wild and cultivated host species, including *V. vinifera*, vinifera hybrids, *V. aestivalis*, and *V. riparia *(Additional file 1: Table S1; Table 2). In contrast, we found five multilocus haplotypes (nos. *41*-*45*) forming a discrete lineage in group B; none of these multilocus haplotypes was found in eastern North America. However, group B does not differ from the eastern US population at all loci (Figure 1; Table 2). It differs for *ITS*/*IGS*, yet shares several haplotypes for *EF1-α *and *TUB2 *with the eastern US population.

### Population differentiation

Differentiation was estimated between the eastern US and introduced populations, and among geographic regions, *Vitis *host habitats, and *Vitis *host species in eastern US. Eastern US and introduced populations are significantly differentiated (Table 4). Within the eastern US, we detected significant differentiation among geographic regions (southeast US, northeast US, and central US). Geographic differentiation within the eastern US was detected when isolates from all hosts were included in the analysis. Because many hosts are more abundant in particular geographic regions (for example, *V. riparia *in the northeast US or *V. rotundifolia *in the southeast US), we conducted tests of geographic subdivision on isolates collected only from *V. vinifera *and vinifera hybrid hosts, which are found abundantly among the three regions, to avoid confounding host and geography. Geographic subdivision was still evident when the analyses only included isolates from *V. vinifera *and vinifera hybrid hosts (Table 4).

**Table 4:** Population structure of *Erysiphe necator *by geographic region and host species.

Populations compared (sample sizes are in parentheses)	*S*_nn_^1^	*H*_ST_^1^
Eastern US (103) vs. introduced (43) populations	**0.9182** **(< 0.001)**	**0.0627** **(< 0.001)**
Within the eastern US		
by geographic region northeast (59) vs. southeast (34) vs. central (10)	**0.6856** **(< 0.001)**	**0.0280** **(< 0.001)**

by geographic region on *Vitis vinifera *and vinifera hybrid hosts northeast (33) vs. southeast (15) vs. central (9)	**0.6384** **(< 0.001)**	**0.0396** **(0.001)**

by host agro-ecological habitat^2^ cultivated (59) vs. wild (34) hosts	0.5733 (0.133)	0.0002 (0.407)

by host species within the northeast US *V. vinifera *(13) vs. vinifera hybrid (20) vs. *V. labrusca *(10) vs. *V. riparia *(8)	0.3532 (0.064)	0.0051 (0.333)

by host species within the southeast US including *V. rotundifolia*^3^ *V. rotundifolia *(5) vs. *V. vinifera *(11) vs. vinifera hybrid (4) vs. *V. labrusca *(3) vs. *V. aestivalis *(9)	**0.3529** **(0.008)**	**0.0857** **(0.007)**

by host species within the southeast US excluding *V. rotundifolia* *V. vinifera *(11) vs. vinifera hybrid (4) vs. *V. labrusca *(3) vs. *V. aestivalis *(9)	0.2552 (0.712)	-0.0179 (0.709)

^1^The nearest neighbour statistic (*S*_nn_) measures the proportion of times the most similar sequence ('nearest neighbour') is from the same population [67]. *H*_ST _is a measure of population subdivision that estimates *F*_ST _among haplotypes [68]. Significant differentiation between or among populations (α = 0.05) is shown in bold. *P*-values are in parentheses.

^2^Isolates from the central US were excluded because all but one were from cultivated species.

^3^A single isolate from *V. riparia *was excluded from the southeast population.

There was no differentiation between populations from wild and cultivated host habitats or among host species within geographic regions (Table 4), except when isolates from *V. rotundifolia *were included in the analyses.

## Discussion

### Patterns of introduction and invasion

The multilocus haplotype network (Figure 2) demonstrates that the eastern US population is ancestral to the introduced populations [32,33]. Therefore, our results are consistent with the hypothesis that *E. necator *was introduced into Europe from eastern North America [11] because populations in Italy and France are derived from North American ancestors. Additionally, populations in California share haplotypes with populations from Europe, suggesting the possibility that they could have been introduced from Europe; the reverse direction of introduction is less likely given the historical records of trade in grapevines and that grape powdery mildew was first observed in Europe in 1845, but not in California until 1859. However, we cannot make any firm conclusions because we do not know which genetic group, A or B, was introduced into Europe first. Populations in the eastern US are more genotypically diverse than those in Europe, Australia and the western US. Several of the haplotypes for individual loci in the introduced populations were found in eastern North America, which is expected when comparing source and introduced populations. An alternative hypothesis, that *E. necator *was introduced into Europe from Japan has been suggested [34], but there is no evidence to support this claim and we were unable to obtain samples from Japan for this study.

At least two haplotypes of the grape powdery mildew fungus, progenitors of groups A and B, were introduced and successfully invaded Europe and Australia. If there had been a single introduction, individuals in the introduced populations would represent a monophyletic group; the single introduction hypothesis can be rejected based on the relationships of group A and B haplotypes in the multilocus network (Figure 2). The introductions, and successful invasions, may have occurred at separate times or multiple, distinct haplotypes may have been introduced during a single event. Previous studies in French and Australian populations of *E. necator*, based on anonymous markers, also found greater diversity in group B than in group A [21,23] and it has been hypothesized that group A is predominantly asexual, while group B undergoes sexual recombination. Most studies have shown that group A is dominated by a single mating type [19,21,23]. We expected to find both genetic groups from the introduced populations in the eastern US population. However, we found isolates in the southeastern US with the same multilocus haplotype as that in group A, but we did not find any with the same haplotype as those in group B. Group B haplotypes may have diverged by genetic drift from the original founders since the first introductions into Europe more than 160 years ago. Sexual reproduction and recombination in group B, coupled with selection for new haplotypes on a different host species and in environmental conditions in Europe, could also have led to divergence. Alternatively, our sample size in eastern North America may not have been large enough to include haplotypes that are less common in the eastern US population, which by chance could have been introduced into Europe. An alternative explanation is that the unique alleles specific to genetic group B came from an entirely different source, which we did not sample for this study.

Genotypic diversity was significantly greater in the eastern US population than in introduced populations, although measures of gene diversity were not always greater (Table 3). In fact, pairwise nucleotide diversity (π) was significantly greater for *ITS*/*IGS *in the introduced population. One explanation for this finding is that the occurrence of two distinct genetic groups in the introduced populations results in high gene diversity because of fixed nucleotide differences between lineages, but low genotypic diversity because there is little or no variation within groups. This discrepancy is similar to finding high gene diversity combined with low genotypic diversity in clonal diploid populations with fixed heterozygosity [35,36]. Multiple introductions of distinct lineages from different sources into new ranges can result in greater diversity than expected during an invasion [4,5]. Additionally, gene diversity was overestimated in Europe and Australia because our samples were not random, but rather were artificially constructed with roughly equal numbers of isolates from the two genetic groups, whereas group B is typically found at a greater frequency than group A in populations in Europe [19,22-25]. Moreover, the lack of diversity within groups A and B validated our strategy of sequencing relatively small samples from Europe and Australia where extensive sampling only found these two discrete groups [19-25]. Haplotypic (or allelic) richness is one of the best measures for reductions in diversity associated with population bottlenecks because rare haplotypes are often lost during founder events even if overall gene diversity is not largely affected [2].

Our results are consistent with historical records of the movement of grapevines and plant-mediated introductions of *E. necator *into Europe, California, and Australia. After grape powdery mildew spread throughout Europe by the mid-1850 s, additional vines were imported from eastern North America as sources of resistance. Unfortunately, this resulted in the introduction of additional grape pests and diseases into Europe, including the phylloxera aphid and downy mildew [37,38]. Additional importations of grapevines from eastern North America for resistance to these pests/diseases may have led also to additional introductions of *E. necator*. Secondary introductions of *E. necator *from Europe into California and Australia are also consistent with historical records of the movement of grapevines. During the 1850's and 1860's large collections of *V. vinifera *were brought to California from Europe [39]. Powdery mildew was first described in California in 1859 and in Australia in 1866 [15,16], so it is likely that it was introduced on vines imported at this time. Two of the four group B multilocus haplotypes found in Europe are also found in the western US. It is not clear why both genetic groups were introduced into Australia, but only group B is present in California. It is possible that group A is present in California, but at such a low frequency that we did not sample it. Nevertheless, it is surprising that since its introduction over 150 years ago, additional genotypes of *E. necator *have not been successfully introduced by the movement of vines from the eastern US to Europe, Australia or the western US.

### Absence of host specificity among *Vitis *host species, except *V. rotundifolia*

With the exception of specialization on muscadine grapes, *V. rotundifolia*, we found no genetic differentiation among populations from *Vitis *host species. This was not unexpected. The best-studied powdery mildew fungus, *Blumeria graminis*, shows specialization among host genera rather than among species within a genus [40,41]. Similarly, *E. necator *demonstrates host specialization at the level of host genus. Gadoury and Pearson [42] showed that *E. necator *var. *ampelopsidis *sampled from *P. quinquefolia *was only rarely pathogenic on *Vitis *species, and then only with low virulence. Multilocus sequencing of *E. necator *var. *ampelopsidis*, as in the formae speciales of *B. graminis*, showed that this type of marked host specialization correlates to marked genetic divergence from *E. necator *on *Vitis*. Alternatively, there may be population divergence of *E. necator *among *Vitis *hosts, but we are not able to detect it with the conserved genes used in this study. Other fungi show specialization at the level of host species. For example, microsatellite markers, which are more polymorphic than multilocus sequences, allowing for better detection of differentiation, showed specialization of *Microbotryum violaceum *at the level of host species [43]. The lack of specialization could be also explained by recent colonization of *Vitis *hosts by *E. necator *or recent diversification of *Vitis *species in North America. In closely related species or populations undergoing speciation, genetic divergence may only be evident at one or a few loci involved in adaptation and reproductive isolation [44].

We found that *E. necator *populations from muscadines are genetically distinct from populations on other *Vitis *species. Although, the haplotypes of muscadine isolates differ from those from other *Vitis *species by one or two mutations there is a strong phenotypic difference that is a potential isolating mechanism. Another study demonstrated marked host specialization to muscadine in laboratory inoculations, but not among other *Vitis *species [45]. Although isolates from muscadines could infect other *Vitis *species in the lab, we did not find haplotypes from the muscadine lineage from other *Vitis *species in the field even when they were sympatric with muscadines. Populations of *E. necator *from muscadine and other *Viti*s species could be in the early stages of speciation resulting from host specialization. Alternatively, muscadine isolates may have alleles that evade recognition by host defences in a gene-for-gene interaction. Resistance to powdery mildew controlled by a single, but complex, genetic locus has been demonstrated in muscadines, which is a source of resistance in breeding programs [46]. It is important to test any new resistant cultivars derived from muscadines with diverse powdery mildew populations from the regions where muscadines are endemic to ensure that the resistance would be durable.

Population differentiation of *E. necator *was not detected between wild and cultivated hosts. In some cases, crop domestication can lead to the divergence of pathogen populations on wild relatives and crop plants [47]. Moreover, management strategies or high-density cultivation of crop plants can lead to population differentiation between pathogens from natural ecosystems and agricultural ecosystems [48]. The lack of population structure in *E. necator *indicates that gene flow is presently occurring or has occurred historically between the powdery mildew populations from wild and cultivated hosts.

## Conclusions

Our results are consistent with the hypotheses that populations of the grape powdery mildew fungus, *E. necator*, in Europe are derived from two separate introductions and that their ancestors were likely from native populations in the eastern US. Multilocus sequencing analysis and historical records are also consistent with the hypothesis that the initial introductions into Europe were followed by secondary introductions from Europe into the western US and Australia and were likely the result of plant-mediated dispersal in the grapevines that were frequently traded between continents during the time of introductions. Within the eastern US, populations of *E. necator *do not demonstrate divergence based on host habitat or *Vitis *host species, with the exception of specialization to muscadine grapes, *V. rotundifolia*.

## Methods

### Grape powdery mildew pathosystem

Powdery mildew fungi are haploid ascomycetes that are obligate parasites of plants that produce colonies of superficial hyphae and asexual spores (conidia). *E. necator *infects *Vitis *species and other members of the Vitaceae. *E. necator *can also reproduce sexually if individuals of both of the two mating types are present [42].

Diverse wild *Vitis *species are found throughout eastern North America [49], with many of the species demonstrating at least some susceptibility to powdery mildew [50]. We sampled *E. necator *from cultivated grapes and from four of the most common wild species: *V. riparia, V. aestivalis, V. labrusca*, and *V. rotundifolia *(Table 1). *V. riparia *is common in colder regions of central and northeastern North America. Both *V. aestivalis *and *V. labrusca *are distributed throughout the northeastern US and the higher elevations in the southeastern US. The muscadine grape, *V. rotundifolia*, which is endemic to and widely distributed throughout the southeastern US, has considerable resistance to powdery mildew [51], and is genetically and morphologically distinct from other *Vitis *spp., such that it is sometimes considered to be in a separate genus, *Muscadinia *[52].

Cultivated varieties grown throughout eastern North America are also diverse. Interspecific hybrids derived from crosses between the European wine grape, *V. vinifera*, and wild American *Vitis *species are common as cultivated vines [53]. Cultivated labrusca hybrids (*i.e*. 'Concord' and 'Niagara'; sometimes referred to as *V. labruscana*) were derived mostly from backcrossing to *V. labrusca *and are grown in colder climates of eastern North America. In contrast to the diversity of hosts in eastern North America, most other major wine-producing regions are dominated by *V. vinifera*, which is native to Eurasia [54] and highly susceptible to *E. necator *[55].

### Sampling, isolate maintenance and DNA extraction

We sampled *E. necator *from the eastern US (northeast, southeast, central) and western US (Additional file 1: Table S1). Most of our sampling from wild host species in the southeast was limited to higher elevations because we were not able to find mildew on wild species other than *V. rotundifolia *at lower elevations of the coastal plain. We speculate that this was due to high temperatures and drought that were not conducive to mildew for several weeks prior to our sampling in 2008. Samples from the eastern US were collected to maximize the diversity of host species and host habitats. Samples from France, Italy and Australia were obtained from collaborators who generously sent genomic DNA from *E. necator *isolates collected from cultivated *V. vinifera *and previously identified as genetic group A or B (Additional file 1: Table S1). In this respect, samples from France, Italy and Australia do not represent random samples but they do reflect the diversity found in each country. Populations of *E. necator *in Europe and Australia have been extensively sampled across broad geographic regions [19-24]. Among these studies, a total of approximately 1000 *E. necator *isolates were genotyped with various markers, and each study demonstrated that populations are structured into two genetic lineages designated as groups A and B. We reasoned that additional sampling was not necessary in Europe and Australia for this study because little genetic diversity had been found within the two lineages despite extensive sampling from different cultivars, years, and times of year. We consider that our sampling represents the diversity of isolates in Europe and Australia since the isolates came from both genetic groups across different regions of France, Italy and Australia (we were not able to obtain DNA from other locations with published reports of previous genotyping). In fact, the isolates we sequenced from Australia were identified as having distinct genotypes [21], representative of the total genetic diversity found there previously. Cultivated *V. vinifera *is the only host plant *E. necator *was sampled from in Europe and Australia because this is the dominant species present in these regions. Therefore, we did not sample from wild species outside of the eastern US.

Isolates of powdery mildew from *Parthenocissus quinquefolia*, also in the Vitaceae, were collected in Ithaca, NY, USA for comparison with powdery mildew from *Vitis *species. Powdery mildew from *P. quinquefolia *is considered variety *ampelopsidis *of *E. necator *[30]. Isolates from *Parthenocissus *species exhibit host specialization, although some can infect *V. vinifera *but with greatly reduced growth compared to isolates from *V. vinifera *[42] .

Mildew isolates were maintained as described by Evans *et al. *[56] on young leaves of *V. vinifera *'Cabernet Sauvignon' grown in a greenhouse. Leaves were surface-sterilized in 0.6% sodium hypochlorite for 1.5 min, rinsed twice with sterile distilled water and air dried in a sterile laminar flow hood. Leaves were kept in Petri dishes containing 20 ml of 2% water agar. Colonies of *E. necator *were initially isolated by touching a mildew colony from an infected leaf to a surface-sterilized leaf. Asexual spores (conidia) from the resulting colonies were transferred 6-12 days later with a sterile pipette tip to another surface-sterilized leaf at least once to rid the colonies of contaminants prior to DNA extraction. Isolates were maintained by transferring to new leaves approximately once per month.

For DNA extraction, conidia and hyphae were collected from colonies 2-3 weeks after inoculation by touching a 1-cm^2 ^piece of office tape (Scotch Tape, 3M) to the colony multiple times until the tape was covered in fungal tissue. The tape was placed in a 1.5 mL microcentrifuge tube with 100 μL of 5% chelex [57,58], vortexed for 30 sec and incubated at 95 °C for 20 min. The solution was vortexed again for 5 sec centrifuged briefly, and the supernatant was removed and used as the DNA template for PCR.

### Multilocus sequencing, sequence alignment, and haplotype network construction

Three nuclear loci were PCR-amplified and sequenced from each isolate. The gene regions we sequenced included: *ITS*/*IGS*, *TUB2*, and *EF1-α*. *ITS *[26] and *TUB2 *[27] had been identified previously in *E. necator*, whereas *IGS *and *EF1-α *were identified in *E. necator *in this study. For *ITS*, we developed primers ITSEnF: 5′-AAGGATCATTACAGAGCGAGAGG-3′ and ITSEnR: 5′-GGATGACCGGACAAAGGTG-3′. For *TUB2*, we designed primers Bt2c: 5′-CAGACTGGCCAATGCGTA-3′ and Bt2d: 5′-AGTTCAGCACCCTCGGTGTA-3′ based on the published sequence (GenBank accession no. AY074934) [27]. We identified the *IGS *region in *E. necator *with the conserved ascomycete primers IGS-12a and NS1R [59], then developed primer IGSEn1: 5′-TTTCGGGGGAAAGCCACCA-3′ to pair with NS1R for improved PCR amplification. *EF1-α *was identified in *E. necator *by designing degenerate primers to conserved regions of *EF1-α *in *Sclerotinia sclerotiorum *(GenBank accession no. DQ471086) and *Botrytis cinerea *(GenBank accession no. DQ471045). We then developed primers EF1-5: 5′-ATAGCGACGATGAGCTGCTT-3′ and EF1-6: 5′-TCGAAAAGGTTTGTTGCAGA-3′ for improved PCR amplification. The PCR reactions for *ITS, IGS*, and *TUB2 *were carried out in a total volume of 25 μL. Reaction components included 2.5 μL of 10 × PCR buffer (Takara Bio, Inc.), 2.5 μL dNTPs, 1.25 μL of 10 μM forward and reverse primers, 0.75 U ExTaq (Takara Bio, Inc.), and 1 μL DNA template. Cycling conditions included an initial denaturation at 95°C for 2 min followed by 35 cycles with a denaturation step at 94.5°C for 1 min, annealing at 56°C for 1 min, extension at 72°C for 1 min, followed by a final extension at 72°C for 5 min. PCR products were purified with QIAquick spin columns (QIAGEN). The PCR reaction for *EF1-α *was carried out in a total volume of 50 μL with all components added at 2 × the volumes used in the reactions for the other loci. Thermal cycling was carried out as described for the other loci. The *EF1-α *PCR products were purified by electrophoresis in a 1% agarose gel, excision of the band and purification with the QIAEX II Gel Extraction Kit (QIAGEN). All DNA fragments were sequenced at the Cornell University Life Sciences Core Laboratories Centre using the Applied Biosystems Automated 3730 DNA Analyzer with Big Dye Terminator chemistry and AmpliTaq-FS DNA Polymerase. All gene regions were sequenced in both directions in at least one isolate. Sequences of *EF1-α, ITS, TUB2 *and *IGS *for haplotype *1 *(Table 2) are deposited in GenBank with accession numbers GQ255471, GQ255473, GQ255475, and GQ255476, respectively. Sequences of *EF1-α, ITS *and *TUB2 *and from *E. necator *var. *ampelopsidis *isolates from *P. quinquefolia *are deposited under accession numbers GQ255474, GQ255470 and GQ255472, respectively.

Sequences were aligned and manually edited in SeqMan (DNASTAR, Inc). Haplotype networks were constructed for each locus and for combined multilocus sequences by statistical parsimony with the program TCS 1.21 [60,61]. Haplotype networks are preferable for intraspecific analyses because they allow for the coexistence of ancestral and derived haplotypes and account for recombination [62]. Alternative, most parsimonious networks are accounted for by this method as loops in the network. The networks were assembled based on an absolute distance matrix between haplotypes, i.e., the number of mutations separating each haplotype, with a parsimony probability of 95%. The ancestral haplotype for each network was predicted based on rooting probability, which assesses the frequency of a particular haplotype and the number of linkages [33]. We also predicted the ancestral haplotype by maximum parsimony using *E. necator *var. *ampelopsidis *as an outgroup. Outgroup haplotypes could not be incorporated into the network with TCS due to high divergence.

### Estimates of diversity and tests of neutrality

To compare diversity between source and introduced populations we estimated several population genetic parameters. They include: haplotype richness (*h*_R_), the total number of haplotypes; Watterson's theta (θ_w_), which is a measure of nucleotide polymorphism equivalent to 2*N*_e_μ (in a haploid population) and an estimate of the effective population size [63]; and π, the pairwise nucleotide diversity [64]. Each parameter was estimated for the eastern US and introduced populations separately using DnaSP v5 [65]. To avoid the bias in diversity estimates caused by differences in sample sizes, we used bootstrapping to conduct rarefaction analysis [66]. For the eastern US population, we sampled a smaller numbers of individuals, with replacement, equal to the sample size of the introduced population and estimated *h*_R_, θ_w_, and π. This was repeated 1000 times and the median estimates for each parameter were recorded. We conducted a one-tailed test to determine if eastern US populations were more diverse than introduced populations. *P*-values were estimated as the proportion of the null distribution that was less than the observed diversity estimate for the introduced population.

Tajima's *D *[31] was calculated by using DnaSP v5 to test for departure from an equilibrium neutral model of evolution. Significant departures from neutrality were determined by permutation tests with 1000 replications.

### Population structure

Differentiation among geographic regions, host habitats, and host species in eastern North America was estimated on combined multilocus sequences. For these analyses labrusca hybrids were grouped with *V. labrusca *rather than the vinifera hybrid group since they are most similar to *V. labrusca *(National Grape Registry http://ngr.ucdavis.edu. The nearest neighbour statistic (*S*_nn_) measures how often the most similar sequence or sequences ('nearest neighbour') is from the same designated population [67]. This statistic was selected for analyses because it has high power with small sample sizes. *S*_nn _estimates the proportion of nearest neighbours that are from the same population versus from a different population. With two populations, for example, a value close to 1 suggests that the two populations are highly differentiated, because almost every sequence would be most similar to other sequences from the same population, whereas a value of 0.5 would be expected if populations are not genetically structured because the closest sequences would be most similar to those from either population with equal probability. We also estimated differentiation with *H*_ST_, a powerful measure of population subdivision that estimates *F*_ST _among haplotypes [68]. Both *S*_nn _and *H*_ST _were calculated by using DnaSP v5. *P*-values were estimated by permutation tests with 1000 replications.

## Authors' contributions

MTB participated in the design of the study, conducted the research, analyzed the data, interpreted the results, and wrote the manuscript. MGM conceived the study, participated in the design of the study, performed some of the analyses, assisted with interpreting the results, and critically revised drafts of the manuscript. Both authors read and approved the final manuscript.

## Supplementary Material

Additional file 1 **Table S1**. Origin, collection date, and multilocus haplotypes of ***Erysiphe necator ***isolates. Click here for file
