# Supplementary material for: Phylogeography and population structure of the grape powdery mildew fungus, Erysiphe necator, from diverse Vitis species
Source: BMC Evol Biol. 2010 Sep 1;10:268. doi: 10.1186/1471-2148-10-268 (PMC2941690; doi:10.1186/1471-2148-10-268)
Supplement: Additional file 1 — Table S1. Origin, collection date, and multilocus haplotypes of Erysiphe necator isolates. [file 1471-2148-10-268-S1.DOC]

| **Table S1: Origin, collection date, and multilocus haplotype of *Erysiphe necator* isolates.** | | | | | |
| --- | --- | --- | --- | --- | --- |
| **Region^1^** | **Location** | **Original *Vitis* Host Species^2^** | **Collection Date** | **Isolate Name** | **Haplotype^3^** |
| SE | Ringgold, GA | *V. rotundifolia* ‘Carlos’ | Sept. 2008 | GAcmus | 35 |
| . | DeSoto Falls, GA | *V. rotundifolia* (w) | Sept. 2008 | GAwmus | 35 |
| . | Blairsville, GA | *V. vinifera* ‘Merlot’ | Sept. 2008 | GAmer1 | 33 |
| . | Blairsville, GA | *V. vinifera* ‘Merlot’ | Sept. 2008 | GAmer2 | 24 |
| . | Blairsville, GA | *V. vinifera* ‘Merlot’ | Sept. 2008 | GAmer3 | 33 |
| . | Blairsville, GA | *V. vinifera* ‘Merlot’ | Sept. 2008 | GAmer4 | 1 |
| . | Blood Mountain, GA | *V. aestivalis* (w) | Sept. 2008 | BlMtnt2 | 4 |
| . | Blood Mountain, GA | *V. aestivalis* (w) | Sept. 2008 | BlMtnS2 | 33 |
| . | Hurdle Mills, NC | *V. rotundifolia* ‘Carlos’ | Sept. 2008 | RoAcmus4 | 35 |
| . | Hurdle Mills, NC | *V. rotundifolia* (w) | Sept. 2008 | RoAwmus2 | 34 |
| . | Hurdle Mills, NC | *V. rotundifolia* (w) | Sept. 2008 | RoAwmus3 | 35 |
| . | Hurdle Mills, NC | *V. vinifera* ‘Cabernet Sauvignon’ | Sept. 2008 | RoACS | 1 |
| . | Hurdle Mills, NC | vinifera hybrid ‘Chardonel’ | Sept. 2008 | RoACl2 | 33 |
| . | Graham, NC | vinifera hybrid ‘Chardonel’ | Sept. 2008 | BenCl2 | 1 |
| . | Graham, NC | vinifera hybrid ‘Seyval Blanc’ | Sept. 2008 | BenS2 | 1 |
| . | Mocksville, NC | *V. vinifera* ‘Cabernet Sauvignon’ | Sept. 2008 | Raylen3 | 12 |
| . | Hamptonville, NC | *V. vinifera* ‘Chardonnay’ | Sept. 2008 | LGNC2 | 11 |
| . | Columbus, NC | *V. vinifera* ‘Cabernet Sauvignon’ | Sept. 2008 | MizeCS1 | 1 |
| . | Tryon, NC | *V. vinifera* ‘Chardonnay’ | Sept. 2008 | RHNC3 | 14 |
| . | Pittsboro, NC | vinifera hybrid ‘Chambourcin’ | Sept. 2008 | SHNC1 | 37 |
| . | Asheville, NC | *V. vinifera* ‘Chardonnay’ | Sept. 2008 | BiltCY2 | 33 |
| . | Pisgah Nat’l Forest, NC | *V. labrusca* (w) | Sept. 2008 | NClab2 | 15 |
| . | Pisgah Nat’l Forest, NC | *V. aestivalis* (w) | Sept. 2008 | NCaes3 | 39 |
| . | Pisgah Nat’l Forest, NC | *V. aestivalis* (w) | Sept. 2008 | NCaes6 | 14 |
| . | Brevard, NC | *V. labrusca* (w) | Sept. 2008 | NClab1 | 25 |
| . | Cashier, NC | *V. labrusca* (w) | Sept. 2008 | NClab5 | 14 |
| . | Cashier, NC | *V. aestivalis* (w) | Sept. 2008 | NCaes1 | 33 |
| . | West Haven, NC | *V. riparia* (w) | Sept. 2008 | NCrip1 | 33 |
| . | Crossville, TN | *V. vinifera* | Sept. 2008 | BSTN4 | 1 |
| . | Blacksburg, VA | *V. aestivalis X riparia* (w) | Oct. 2008 | vir1 | 33 |
| . | Plummer, VA | *V. aestivalis* (w) | Sept. 2007 | W10 | 9 |
| . | Plummer, VA | *V. aestivalis* (w) | Sept. 2007 | W16 | 8 |
| . | Sugar Grove, VA | *V. aestivalis* (w) | Sept. 2007 | W11 | 1 |
| . | Sugar Grove, VA | *V. aestivalis* (w) | Sept. 2007 | W12 | 1 |
| C | Waverly, MO | vinifera hybrid ‘Norton’ | Sept. 2008 | WVMONt | 16 |
| . | Ste. Genevieve, MO | *V. vinifera* ‘Syrah’ | Sept. 2008 | SGMOS | 18 |
| . | Ste. Genevieve, MO | vinifera hybrid ‘Chambourcin’ | Sept. 2008 | SGMOChn | 20 |
| . | Ste. Genevieve, MO | *V. vinifera* ‘Viognier’ | Sept. 2008 | SGMOV | 19 |
| . | St. James, MO | vinifera hybrid ‘Noiret’ | Sept. 2008 | SJMONr | 11 |
| . | Purdy, MO | vinifera hybrid ‘Chambourcin’ | Oct. 2008 | PUMOChn | 19 |
| . | Emporia, KS | vinifera hybrid ‘Frontenac’ | Sept. 2008 | KSFron | 38 |
| . | Lawrence, KS | *V. riparia* (w) | Oct. 2008 | kan1 | 17 |
| . | Comanche, TX | *V. vinifera* ‘Cabernet Sauvignon’ | Oct. 2008 | TXCS1 | 23 |
| . | Comanche, TX | *V. vinifera* ‘Cabernet Sauvignon’ | Oct. 2008 | TXCS2 | 23 |
| NE | Geneva, NY | vinifera hybrid ‘Rosette’ | June 2007 | G1 | 10 |
| . | Geneva, NY | vinifera hybrid ‘Rosette’ | June 2007 | G2 | 8 |
| . | Geneva, NY | vinifera hybrid ‘Rosette’ | June 2007 | G3 | 8 |
| . | Geneva, NY | vinifera hybrid ‘Rosette’ | June 2007 | G4 | 3 |
| . | Geneva, NY | vinifera hybrid ‘Rosette’ | June 2007 | G6 | 8 |
| . | Geneva, NY | vinifera hybrid ‘Rosette’ | June 2007 | G7 | 2 |
| . | Geneva, NY | vinifera hybrid ‘Rosette’ | June 2007 | G8 | 5 |
| . | Geneva, NY | vinifera hybrid ‘Rosette’ | June 2007 | G9 | 22 |
| . | Geneva, NY | vinifera hybrid ‘Rosette’ | June 2007 | G12 | 6 |
| . | Geneva, NY | vinifera hybrid ‘Rosette’ | Sept. 2007 | G13 | 11 |
| . | Geneva, NY | vinifera hybrid ‘Rosette’ | Sept. 2007 | G14 | 22 |
| . | Geneva, NY | vinifera hybrid ‘Rosette’ | Sept. 2007 | G15 | 1 |
| . | Geneva, NY | vinifera hybrid ‘Rosette’ | Sept. 2007 | G17 | 10 |
| . | Geneva, NY | vinifera hybrid ‘Rosette’ | Sept. 2007 | G18 | 5 |
| . | Geneva, NY | vinifera hybrid ‘Rosette’ | Sept. 2007 | G19 | 10 |
| . | Geneva, NY | vinifera hybrid ‘Rosette’ | Sept. 2007 | G20 | 10 |
| . | Geneva, NY | vinifera hybrid ‘Rosette’ | Sept. 2007 | G21 | 6 |
| . | Geneva, NY | vinifera hybrid ‘Rosette’ | Sept. 2007 | G22 | 10 |
| . | Geneva, NY | labrusca hybrid ‘Concord’ | Sept. 2007 | GCON1 | 6 |
| . | Geneva, NY | labrusca hybrid ‘Concord’ | Sept. 2007 | GCON2 | 10 |
| . | Geneva, NY | labrusca hybrid ‘Concord’ | Sept. 2007 | GCON3 | 5 |
| . | Ithaca, NY | *V. vinifera* ‘Cabernet Franc’ | May 2007 | En07-3 | 21 |
| . | Watkins Glen, NY | labrusca hybrid ‘Niagara’ | Sept. 2008 | lw1 | 26 |
| . | Watkins Glen, NY | *V. vinifera* ‘Cabernet Franc’ | Sept. 2008 | lw4 | 1 |
| . | Watkins Glen, NY | *V. vinifera* ‘Cabernet Sauvignon’ | Sept. 2008 | lw5 | 2 |
| . | Dresden, NY | *V. vinifera* ‘Chardonnay’ | Sept. 2003 | 10-18 | 11 |
| . | Dresden, NY | *V. vinifera* ‘Chardonnay’ | Sept. 2003 | 10-36 | 1 |
| . | Dresden, NY | *V. vinifera* ‘Chardonnay’ | Sept. 2008 | Dresden2 | 2 |
| . | Fredonia, NY | labrusca hybrid ‘Concord’ | Sept. 2007 | Fcon1 | 25 |
| . | Fredonia, NY | labrusca hybrid ‘Concord’ | Sept. 2007 | Fcon2 | 25 |
| . | Lockport, NY | *V. vinifera* ‘Merlot’ | Oct. 2008 | LNYM | 28 |
| . | Lockport, NY | labrusca hybrid ‘Niagara’ | Oct. 2008 | LNYN | 30 |
| . | Burt, NY | *V. vinifera* ‘Cabernet Sauvignon’ | Oct. 2008 | BNYCS | 9 |
| . | Burt, NY | labrusca hybrid ‘Niagara’ | Oct. 2008 | BNYN | 8 |
| . | Highland, NY | *V. vinifera* ‘Chardonnay’ | Oct. 2008 | HVLCY | 36 |
| . | Highland, NY | *V. vinifera* ‘Lemberger’ | Oct. 2008 | HVLL | 26 |
| . | Athens, NY | vinifera hybrid ‘Pinard’ | Oct. 2008 | ANYP | 1 |
| . | Riverhead, NY | *V. vinifera* ‘Chardonnay’ | Oct. 2008 | LICY | 12 |
| . | Riverhead, NY | vinifera hybrid ‘Norton’ | Oct. 2008 | LINt | 29 |
| . | Ithaca, NY | *V. riparia* (w) | June 2007 | W1 | 1 |
| . | Ithaca, NY | *V. riparia* (w) | June 2007 | W2 | 6 |
| . | Ithaca, NY | *V. riparia* (w) | Aug. 2007 | W6 | 11 |
| . | Ulysses, NY | *V. riparia* (w) | Sept. 2008 | Ith6 | 7 |
| . | Bath, NY | *V. riparia* (w) | Sept. 2007 | Bath | 9 |
| . | Salamanca, NY | *V. riparia* (w) | Sept. 2007 | Sal | 1 |
| . | Watertown, NY | *V. riparia* (w) | Aug. 2008 | Watertown | 2 |
| . | Stemlersville, PA | *V. aestivalis* (w) | Aug. 2008 | sp1 | 9 |
| . | Tompkins Twp, MI | *V. riparia* (w) | Sept. 2008 | MI3 | 8 |
| . | Tompkins Twp, MI | *V. riparia* (w) | Sept. 2008 | MI5 | 1 |
| . | East Brunswick, NJ | *V. labrusca* (w) | Sept. 2008 | EBrunNJ | 32 |
| . | North Haven, CT | *V. aestivalis* (w) | Sept. 2008 | NHCT | 40 |
| . | Lexington, MA | *V. labrusca* (w) | Oct. 2008 | mas1 | 31 |
| . | Lexington, MA | *V. labrusca* (w) | Oct. 2008 | mas4 | 31 |
| . | Bradford, NH | *V. labrusca* (w) | Oct. 2008 | mas2 | 27 |
| . | Chatsworth, NJ | *V. vinifera* ‘Cabernet Franc’ | Oct. 2006 | NJa | 1 |
| . | Barrington, NJ | *V. aestivalis* (w) | Sept. 2007 | Bar | 9 |
| . | Harpersfield, OH | *V. vinifera* | Sept. 2007 | Hv | 13 |
| . | Madison, OH | labrusca hybrid ‘Concord’ | Sept. 2007 | GRcon | 9 |
| . | Geneva, OH | *V. riparia* (w) | Sept. 2007 | GSPk | 8 |
| W | Madera Cty, CA | *V. vinifera* ‘Carignan’ | June 2008 | Ma1A3c | 41 |
| . | Madera Cty, CA | *V. vinifera* ‘Carignan’ | June 2008 | Ma1D2c | 41 |
| . | Madera Cty, CA | *V. vinifera* ‘Carignan’ | June 2008 | Ma2A1c | 41 |
| . | Monterrey Cty, CA | *V. vinifera* ‘Chardonnay’ | May 2007 | CAMont | 43 |
| . | Monterrey Cty, CA | *V. vinifera* ‘Chardonnay’ | June 2008 | MoA1L | 41 |
| . | Monterrey Cty, CA | *V. vinifera* ‘Chardonnay’ | June 2008 | MoC2L | 41 |
| . | Monterrey Cty, CA | *V. vinifera* ‘Chardonnay’ | June 2008 | MoE2L | 41 |
| . | Delta, CA | *V. vinifera* ‘Chardonnay’ | June 2007 | CADelta | 41 |
| . | Davis, CA | *V. vinifera* ‘Red Globe’ | June 2007 | CADavis | 41 |
| . | Paso Robles, CA | *V. vinifera* | Aug. 2007 | CAPr | 41 |
| . | Dundee, OR | *V. vinifera* ‘Pinot Noir’ | Sept. 2008 | ORAS | 41 |
| . | Peoria, OR | *V. vinifera* ‘Muscat’ | Sept. 2008 | ORMUS | 41 |
| . | Corvallis, OR | *V. vinifera* ‘Pinot Noir’ | Sept. 2008 | ORPN | 41 |
| EU | Latresne, France | *V. vinifera* ‘Merlot’ | May 2003 | BCCLAT12 (M.-F. Corio-Costet)^4^ | 42 |
| . | Avignon, France | *V. vinifera* ‘Carignan’ | May 2004 | ACCPVR43 | 33 |
| . | Assas, France | *V. vinifera* ‘Carignan’ | Apr. 1999 | A53 (J.P. Péros) | 33 |
| . | Faugères, France | *V. vinifera* | Jul. 2000 | A232 | 33 |
| . | Faugères, France | *V. vinifera* | Sept. 2001 | B237 | 41 |
| . | Montpellier, France | *V. vinifera* ‘Carignan’ | Apr. 1999 | A44 | 33 |
| . | Montpellier, France | *V. vinifera* ‘Carignan’ | Apr. 1999 | B27 | 41 |
| . | Montpellier, France | *V. vinifera* ‘Carignan’ | Apr. 2000 | B135 | 41 |
| . | Montefiridolfi, Italy | *V. vinifera* ‘Chardonnay’ | June 1998 | T1 (P. Cortesi) | 33 |
| . | Montefiridolfi, Italy | *V. vinifera* ‘Chardonnay’ | June 1998 | T2 | 33 |
| . | Montefiridolfi, Italy | *V. vinifera* ‘Chardonnay’ | June 1998 | T3 | 33 |
| . | Montefiridolfi, Italy | *V. vinifera* ‘Malvasia’ | June 1998 | T6 | 43 |
| . | Montefiridolfi, Italy | *V. vinifera* ‘Malvasia’ | June 1998 | T7 | 43 |
| . | Montefiridolfi, Italy | *V. vinifera* ‘Chardonnay’ | June 2001 | T4 | 33 |
| . | Montalcino, Italy | *V. vinifera* ‘Sangiovese’ | June 1997 | T15 | 41 |
| . | Montalcino, Italy | *V. vinifera* ‘Sangiovese’ | June 1997 | T16 | 41 |
| . | Montalcino, Italy | *V. vinifera* ‘Sangiovese’ | June 1998 | T14 | 33 |
| . | Montalcino, Italy | *V. vinifera* ‘Sangiovese’ | June 1998 | T18 | 41 |
| . | Voghera, Italy | *V. vinifera* | June 2001 | T21 | 33 |
| . | Voghera, Italy | *V. vinifera* | June 2003 | T22 | 33 |
| . | Trinitapoli, Italy | *V. vinifera* ‘Pallieri’ | May 2002 | Am221 (F. Faretra and M. Miazzi) | 33 |
| . | Campomarino, Italy | *V. vinifera* ‘Pinot Blanc’ | May 2002 | Am288 | 33 |
| . | Campomarino, Italy | *V. vinifera* ‘Pinot Blanc’ | July 2002 | Bm316 | 44 |
| . | Guglionesi, Italy | *V. vinifera* ‘Chardonnay’ | Aug. 2002 | Bm329 | 44 |
| AU | Adelaide Plains, Australia | *V. vinifera* | Jan. 1993 | APc1 (E.S. Scott and B.E. Stummer) | 33 |
| . | Barossa Valley, Australia | *V. vinifera* | Jan. 1993 | BNb2 | 33 |
| . | Adelaide Hills, Australia | *V. vinifera* ‘Chardonnay’ | Mar. 1993 | AHd2 | 45 |
| . | Adelaide Hills, Australia | *V. vinifera* ‘Chardonnay’ | Feb. 1993 | AHe1 | 45 |
| . | Adelaide Hills, Australia | *V. vinifera* ‘Sauvignon Blanc’ | Jan. 1993 | AHa1 | 45 |
| . | McLaren Vale, Australia | *V. vinifera* | Jan. 1993 | MVb2 | 45 |
| ^1^regional designations for isolates are SE = southeast US, C = central US, NE = northeast US, W = western US, EU = Europe, AU = Australia.  ^2^(w) indicates that the isolate was collected from a wild *Vitis* species. Vinifera hybrid indicates a cultivated variety derived from interspecific crosses between *V. vinifera* and wild American *Vitis* spp., whereas labrusca hybrid indicates a variety derived from *V. labrusca*.  ^3^Haplotype designation for each isolate corresponds with those defined in Table 2.  ^4^Provider(s) of DNA is listed in parentheses. All isolates below, until the next listed researcher(s), were provided by the same group. | | | | | |
